# Phosphoribosyl transferase domain containing 1: A prognostic biomarker in testicular germ cell tumors

**DOI:** 10.1016/j.omton.2025.200958

**Published:** 2025-02-28

**Authors:** Peisheng Huang, Yihao Chen, Yongcheng Shi, Chuanfan Zhong, Huawei Lin, Xiaoxue Yu, Kai Chen, Zhuoya Huang, Le Zhang, Shumin Fang, Jianming Lu, Jiahong Chen

**Affiliations:** 1 The First Clinical Medical College, Guangdong Medical University, Zhanjiang, Guangdong 524023, China; 2 Department of Urology, Huizhou Central People’s Hospital, Huizhou, Guangdong 516001, China; 3 Department of Urology, Zhujiang Hospital, Southern Medical University, Guangzhou, Guangdong 510282, China; 4 The Second Clinical College of Guangzhou Medical University, Guangzhou, Guangdong 510180, China; 5 Department of Pathology, Guangzhou Medical University Affiliated Women and Children′s Medical Center, Guangzhou 510623, China; 6 Department of Pathology, Huizhou Central People’s Hospital, No. 41, Eling North Road, Huizhou, Guangdong 516001, China; 7 Institute for Integrative Genome Biology, University of California, Riverside, CA 92507, USA; 8 Science Research Center, Huizhou Central People’s Hospital, Huizhou, Guangdong 516001, China; 9 Department of Andrology, Guangzhou First People’s Hospital, Guangzhou Medical University, Guangzhou 510180, China

**Keywords:** MT: Regular Issue, testicular germ cell tumor, biomarker, PRTFDC1, prognosis, immunotherapy

## Abstract

Due to the heterogeneity and complex classification of testicular germ cell tumors (TGCTs), prognostic evaluation and therapeutic targets remain unclear. Therefore, identifying a novel biomarker to comprehensively assess TGCT prognosis and immunotherapy response is crucial. We collected data from 457 TGCT patient samples and 12 normal testicular samples across six cohorts. Differential expression analysis combined with univariate Cox regression identified prognostic markers for TGCT. Multivariate Cox regression and survival analysis further evaluated the prognostic value of phosphoribosyl transferase domain containing 1 (PRTFDC1). Immunohistochemistry on tissue microarrays validated PRTFDC1’s predictive value in clinical samples. We then investigated the relationship between PRTFDC1 and somatic mutations, copy number variations, immune cell infiltration, and immunotherapy response. Through these analyses, we identified PRTFDC1 as an independent risk factor indicating poor prognosis in TGCT. Immunohistochemistry demonstrated high PRTFDC1 expression in TGCT tissues. Gene set enrichment analysis revealed that PRTFDC1 suppresses immune-related pathways. Immune infiltration showed that high PRTFDC1 expression is associated with low CD8^+^ T cell infiltration. Immunotherapy response analysis indicated that low PRTFDC1 expression predicts better immunotherapy response and favorable prognosis. In conclusion, this study elucidates the biological and clinical significance of PRTFDC1, suggesting it as an effective and reliable biomarker for predicting TGCT prognosis and immunotherapy response.

## Graphical abstract

**Figure:**
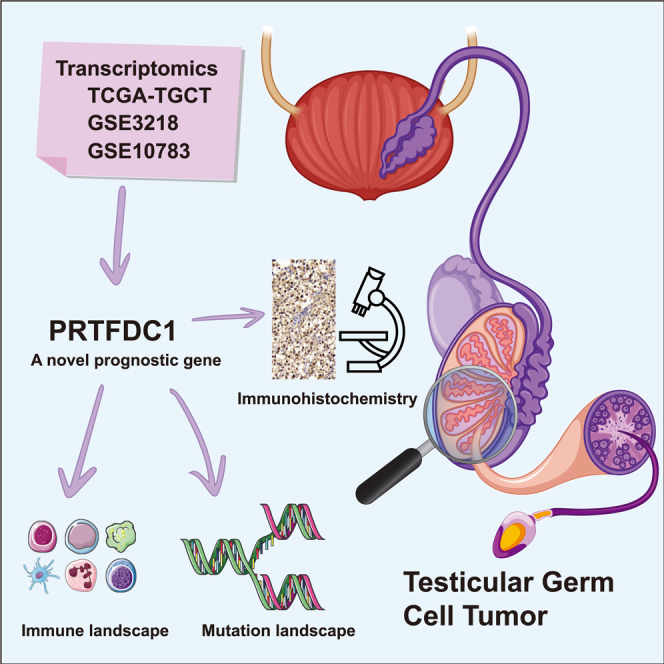

## Teaser

Chen and colleagues report that high expression of PRTFDC1 is frequently observed in testicular germ cell tumors and correlates with poorer prognosis. Patients with testicular germ cell tumors exhibiting low PRTFDC1 expression show greater sensitivity to immunotherapy, highlighting its potential as a prognostic biomarker and therapeutic target.

## Introduction

Testicular germ cell tumors (TGCTs) are among the most common tumors in adolescents and young men aged 15 to 39.^1^ Although the 5-year survival rate for TGCT exceeds 94%, the incidence of new cases has been increasing over the past few decades.^2^ The development of TGCT may be associated with various risk factors, including cryptorchidism, hypospadias, a family history of TGCT, testicular dysgenesis syndrome, and the influence of endogenous and exogenous estrogens.^3^ Additionally, external environmental factors such as diagnostic radiation exposure to the lower abdomen and pelvis are also believed to increase the risk of TGCT.^4^ TGCT originates from germ cells and can present various histological types, such as seminomas, non-seminomas, and mixed TGCT. Seminomas are the most common type, accounting for approximately 50% of all cases. Non-seminomas primarily include embryonal carcinoma, teratoma, yolk sac tumor, choriocarcinoma, and trophoblastic tumor.^5^ Treatment for TGCT varies based on histological type and tumor extent, including surgery, chemotherapy, radiation therapy, or a combination of these.^6^ Seminomas are highly sensitive to radiation and chemotherapy, which correlates with their relatively favorable prognosis. In contrast, non-seminomas and mixed TGCTs have a poorer prognosis due to their diverse tissue components. For instance, teratomas are highly resistant to radiation and chemotherapy, while choriocarcinomas tend to invade blood vessels and can metastasize extensively even when the primary tumor is not prominent.

Currently, clinical prognosis and monitoring of TGCT recurrence and metastasis primarily rely on serum tumor markers and the International Germ Cell Cancer Collaborative Group (IGCCCG) prognosis model. Serum tumor markers include alpha-fetoprotein (AFP), beta-human chorionic gonadotropin (β-HCG), and lactate dehydrogenase (LDH).^7^ Due to the heterogeneous expression of serum biomarkers across different subtypes of TGCTs, these biomarkers exhibit relatively low sensitivity and specificity in tumor detection.^7^ In seminomas, the elevated levels of AFP, β-HCG, and LDH are observed in 2.8%, 28%, and 29.1% of cases, respectively, whereas in non-seminomatous germ cell tumors, the elevated levels of these biomarkers are found in 60.1%, 53.0%, and 38.7% of cases, respectively.^8^ AFP and β-HCG are primarily elevated in specific components of non-seminomatous germ cell tumors.^8^ Additionally, elevated levels of AFP and β-HCG may also occur in other diseases, potentially leading to false-positive results.^9^^,^^10^ LDH is widely expressed in both malignant and non-malignant cells, which reduces its specificity as a tumor marker.^11^ The IGCCCG prognosis model classifies patients into good, intermediate, and poor prognosis groups based on a comprehensive assessment of histological type, primary site, metastatic sites, and serum tumor markers, guiding standard treatment and first-line chemotherapy choices.^12^ However, this model has limitations. It may not be applicable to all patients, especially those with rare types of TGCT or those who fail first-line chemotherapy, as it may not fully reflect individual patient conditions.^13^ Therefore, in the context of precision medicine, identifying prognostic biomarkers with higher sensitivity and specificity has become urgent and necessary for TGCT research.

This study utilized differential analysis, univariate and multivariate Cox regression analysis, and immunohistochemistry (IHC) to identify Phosphoribosyl Transferase Domain Containing 1 (PRTFDC1) as a potential prognostic biomarker for TGCT patients. Further analysis explored the potential functions, mutation characteristics, and drug target potential of PRTFDC1. Furthermore, we found that PRTFDC1 could predict patients' potential response to immunotherapy.

## Results

### Identification and functional enrichment analysis of differentially expressed genes

**Figure 1:**
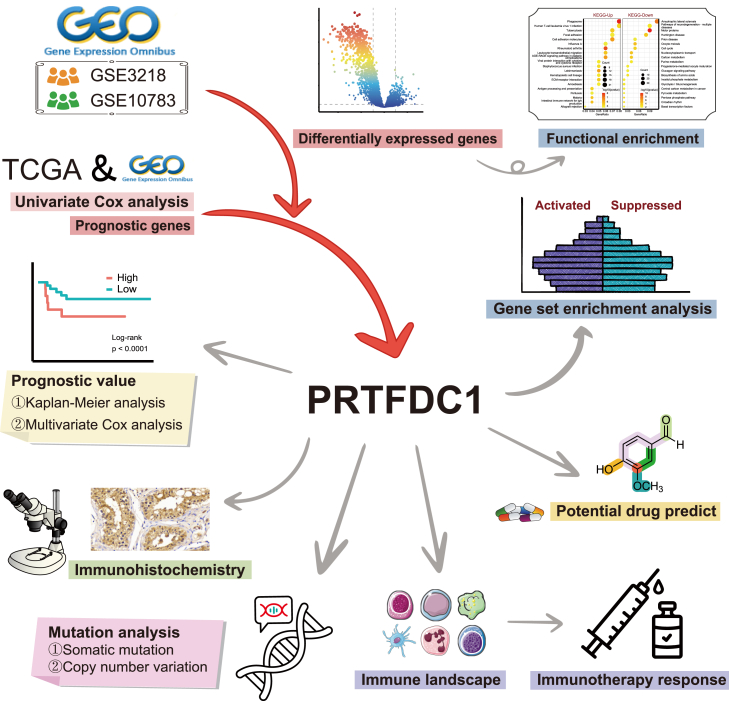
Flowchart of this study

**Figure 2:**
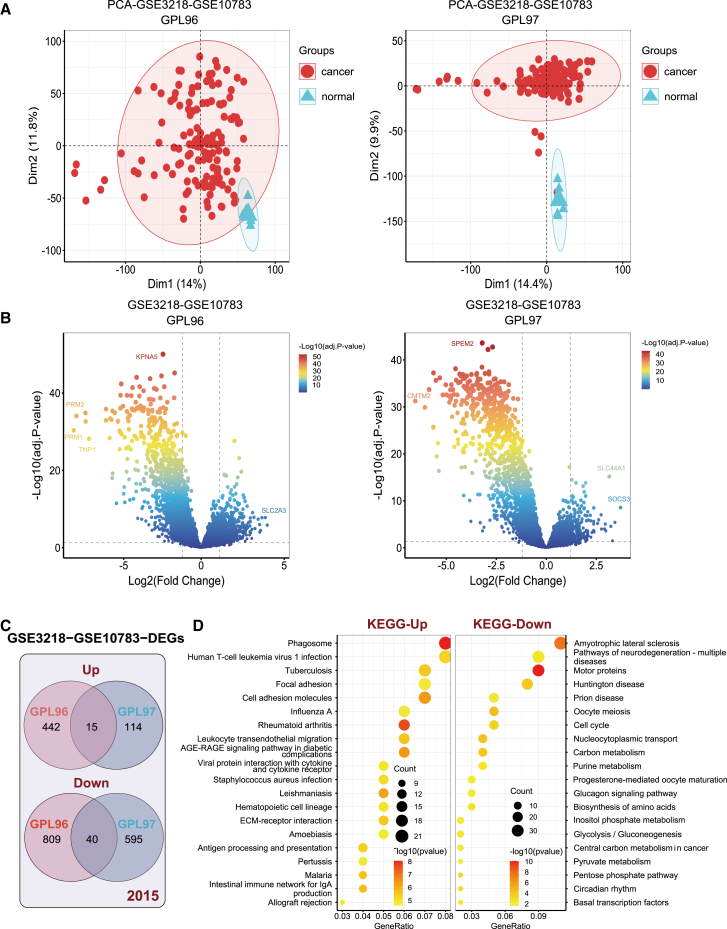
Identification and functional enrichment analysis of differentially expressed genes between normal testicular tissue and TGCT (A) Principal components analysis results of the GSE3218-10783 (GPL96, GPL97) cohort. (B) Volcano plot of differentially expressed genes between normal testicular tissue and TGCT. (C) Venn diagram of upregulated and downregulated differentially expressed genes between the GPL96 and GPL97 platforms. (D) Kyoto Encyclopedia of Genes and Genomes (KEGG) enrichment analysis results of upregulated and downregulated differentially expressed genes.

The workflow of this study is illustrated in Figure 1. Initially, we performed differential expression analysis on two merged cohorts to identify differentially expressed genes (Figures 2A and 2B). In the GSE3218-10783 (GPL96) cohort, 1,306 differentially expressed genes were identified, with 457 upregulated and 849 downregulated. In the GSE3218-10783 (GPL97) cohort, 764 differentially expressed genes were identified, with 129 upregulated and 635 downregulated. To achieve comprehensive differential gene expression screening, we integrated the differentially expressed genes from GSE3218-10783 (GPL96) and GSE3218-10783 (GPL97) using a union strategy. This approach resulted in the identification of 2,015 differentially expressed genes, comprising 571 upregulated and 1,444 downregulated genes (Figure 2C).

We then utilized the "clusterProfiler" R package to perform Kyoto Encyclopedia of Genes and Genomes (KEGG) enrichment analysis on the upregulated and downregulated genes separately, to further explore their functions in TGCTs. The results indicated that the upregulated genes were primarily enriched in pathways such as phagosome, focal adhesion, cell adhesion molecules, extracellular matrix (ECM)-receptor interaction, and antigen processing and presentation. Conversely, the downregulated genes were enriched in pathways including cell cycle, glycolysis/gluconeogenesis, central carbon metabolism in cancer, and purine metabolism (Figure 2D). These findings suggest that these differentially expressed genes may influence cell division, metabolism, adhesion, and extracellular matrix interactions, thereby promoting TGCT malignancy. Specifically, the focal adhesion and cell adhesion molecule pathways may promote TGCT growth and metastasis through mechanisms such as tumor angiogenesis and increased vascular permeability.^14^^,^^15^ Activation of the ECM-receptor interaction pathway could facilitate interactions between tumor cells and the tumor microenvironment, contributing to TGCT cell survival and dissemination.^16^ Downregulation of cell-cycle-related genes may lead to dysregulated cell-cycle control by reducing the expression of key inhibitors such as p53 and RB, thereby promoting uncontrolled proliferation of TGCT cells.^17^

### PRTFDC1, a differentially expressed gene tightly associated with prognosis

**Figure 3:**
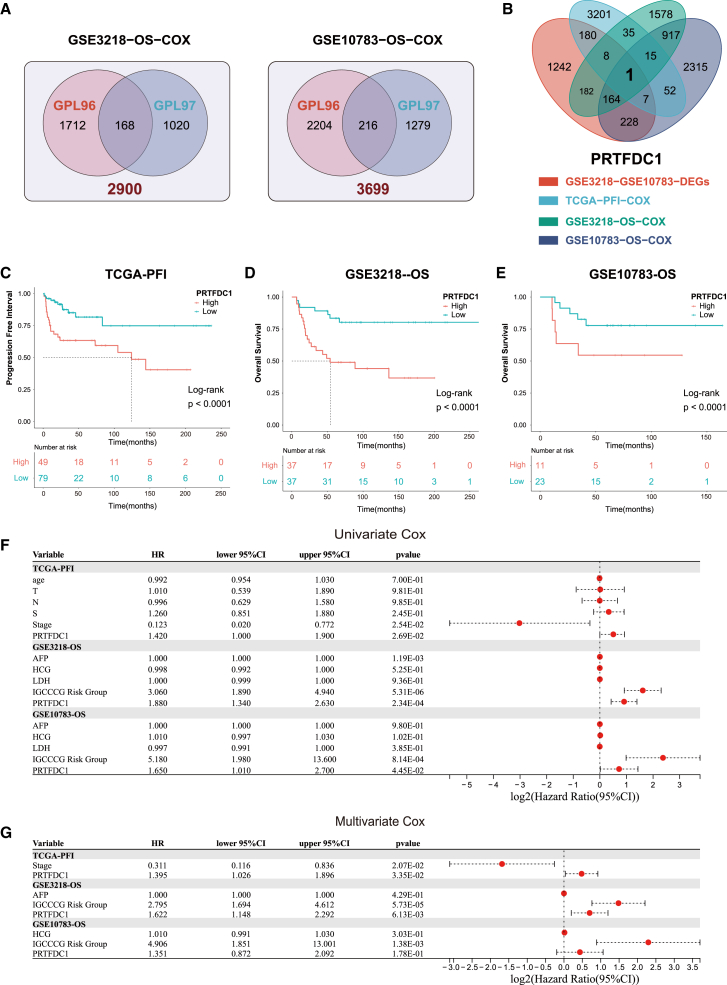
Identification and prognostic value assessment of PRTFDC1 (A) Venn diagram of prognostic genes between the GPL96 and GPL97 platforms in the GSE3218 and GSE10783 datasets, considering overall survival (OS) as the outcome event. (B) Venn diagram of differentially expressed genes and prognostic genes. (C–E) Kaplan-Meier survival analysis for all cohorts with progression-free interval (PFI) and overall survival (OS) as outcome events. (F) Univariate Cox regression analysis of clinical characteristics, including PRTFDC1, age, and tumor stage, across all cohorts. (G) Multivariate Cox regression analysis of PRTFDC1 across all cohorts.

Based on univariate Cox regression analysis, we identified 10,482 prognostic genes in The Cancer Genome Atlas (TCGA) cohort and four Gene Expression Omnibus (GEO) cohorts. We then employed a union strategy to merge the prognostic genes identified from the GEO datasets (GPL96 and GPL97) to achieve a comprehensive screening (Figure 3A). Specifically, GSE3218 identified 2,900 prognostic genes, GSE10783 identified 3,699 prognostic genes, and TCGA cohort identified 3,499 prognostic genes. As shown in Figure 3B, we identified a differentially expressed gene closely associated with prognosis (PRTFDC1).

To validate the impact of PRTFDC1 on TGCT prognosis, we conducted Kaplan-Meier survival analysis. The results indicated that the high PRTFDC1 expression group had significantly poorer prognosis compared with the low expression group (Figures 3C–3E). Univariate and multivariate Cox regression analyses indicated that PRTFDC1 could serve as an independent risk factor for progression-free interval (PFI) or overall survival (OS) in the TCGA-TGCT and GSE3218 cohorts. Although the multivariate Cox analysis in the GSE10783 cohort did not show significant statistical difference (*p* = 0.18), the data suggested a trend (hazard ratio [HR] = 1.351), indicating that PRTFDC1 might be an independent prognostic predictor (Figures 3F and 3G). Thus, based on these results, PRTFDC1 could be a potential biomarker for predicting the prognosis of TGCT patients. The IGCCCG risk group, shown in Figures 3F and 3G, is an important independent risk factor associated with OS. To better facilitate clinical translation, we combined the IGCCCG risk group and PRTFDC1 expression to construct a nomogram (Figure S2A). The calibration curve demonstrates the reliability of our nomogram in predicting OS (Figure S2B).

### Biological function analysis of PRTFDC1

**Figure 4:**
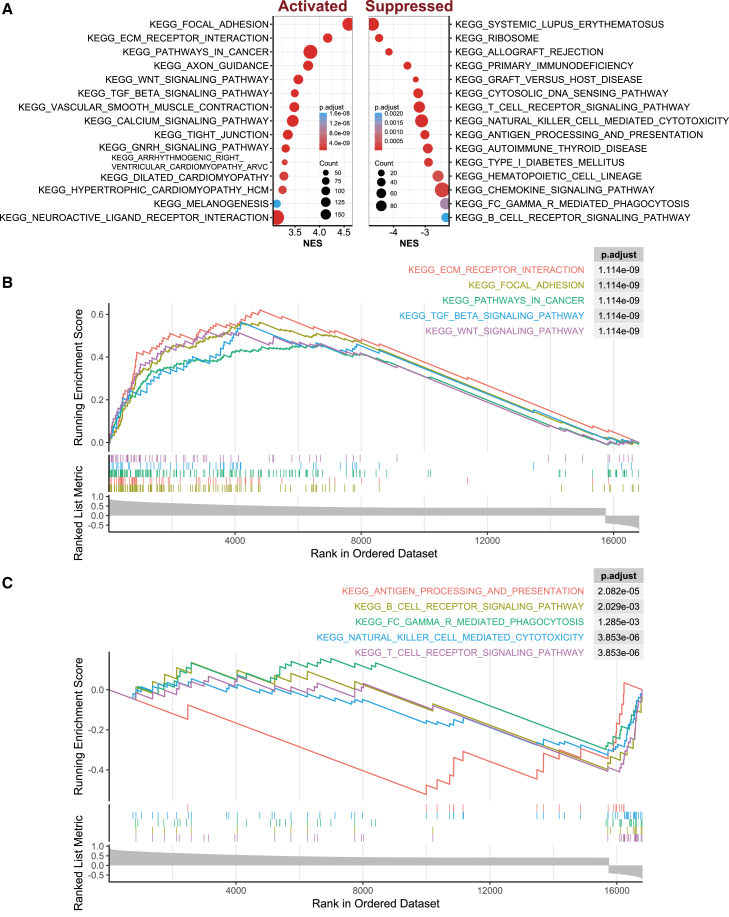
Gene set enrichment analysis (A) GSEA results related to PRTFDC1 in the TCGA cohort. (B) Pathways associated with tumorigenesis, development, and metastasis in the activated pathways. (C) Immune-related pathways in the suppressed pathways.

We performed Gene Set Enrichment Analysis (GSEA) to investigate the biological characteristics of PRTFDC1 (Figure 4A). Based on normalized enrichment scores (NES), we identified the top 15 activated and suppressed pathways. Notably, we observed the activation of pathways related to tumor progression (Figure 4B), such as focal adhesion, ECM-receptor interaction, pathways in cancer, transforming growth factor (TGF)-beta signaling pathway, and WNT signaling pathway. Conversely, several immune-related pathways were suppressed (Figure 4C), including antigen processing and presentation, B cell receptor signaling pathway, Fc gamma R-mediated phagocytosis, natural killer cell-mediated cytotoxicity, and T cell receptor signaling pathway. These results suggest that PRTFDC1 may promote TGCT malignancy by activating pathways involved in tumor cell proliferation and suppressing processes related to antigen presentation and immune cell activation.

### High expression of PRTFDC1 protein in TGCT tissues

**Figure 5:**
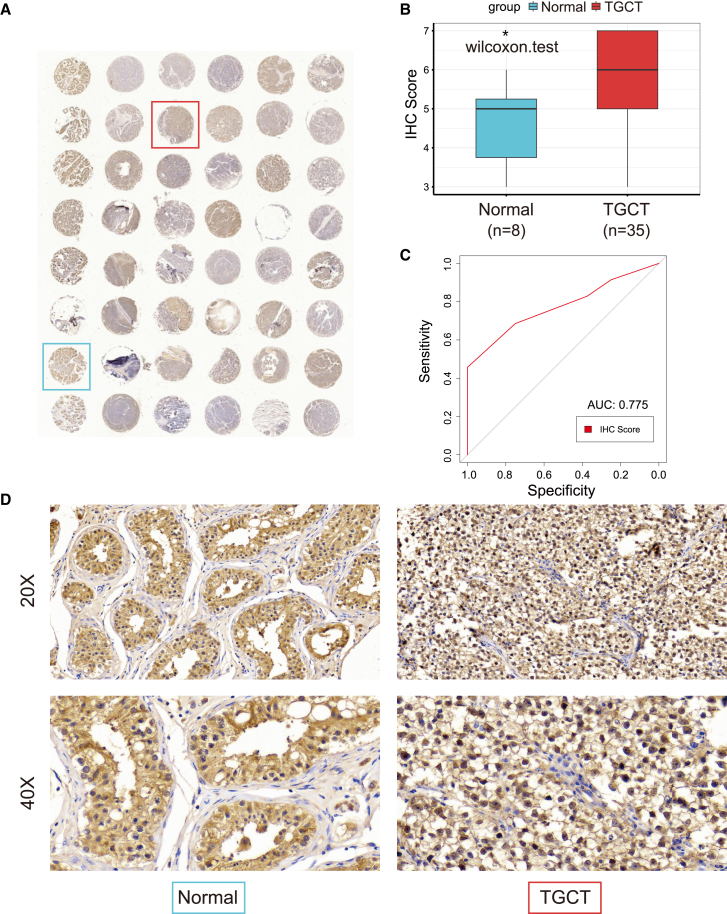
External validation of high PRTFDC1 expression in TGCT (A) IHC staining of PRTFDC1 in 48 clinical samples from tissue microarrays. (B) Statistical analysis of PRTFDC1 IHC scores in normal testicular tissue (*n* = 8) and TGCT (*n* = 35) samples. (C) ROC AUC of PRTFDC1. (D) Representative IHC staining images of PRTFDC1 in normal testicular tissue and TGCT.

To investigate the expression of PRTFDC1 in TGCT tissues, we analyzed 48 human testicular tissue microarrays, excluding five cases of B cell lymphoma. We employed IHC to detect PRTFDC1 protein levels and subcellular localization in 35 TGCT tissues and eight normal testicular tissues (Figure 5A). The results showed that PRTFDC1 was highly expressed in TGCT tissues compared with normal testicular tissues and was primarily localized in the nucleus and cytoplasm (Figure 5D). Immunohistochemical scoring of these 43 tissue samples revealed that the tumor group had significantly higher scores than the normal group (Figure 5B). Furthermore, receiver operating characteristic (ROC) curve analysis demonstrated that PRTFDC1 had good predictive performance in clinical samples (area under the curve [AUC] = 0.775; Figure 5C).

### Mutation landscape of TGCT

**Figure 6:**
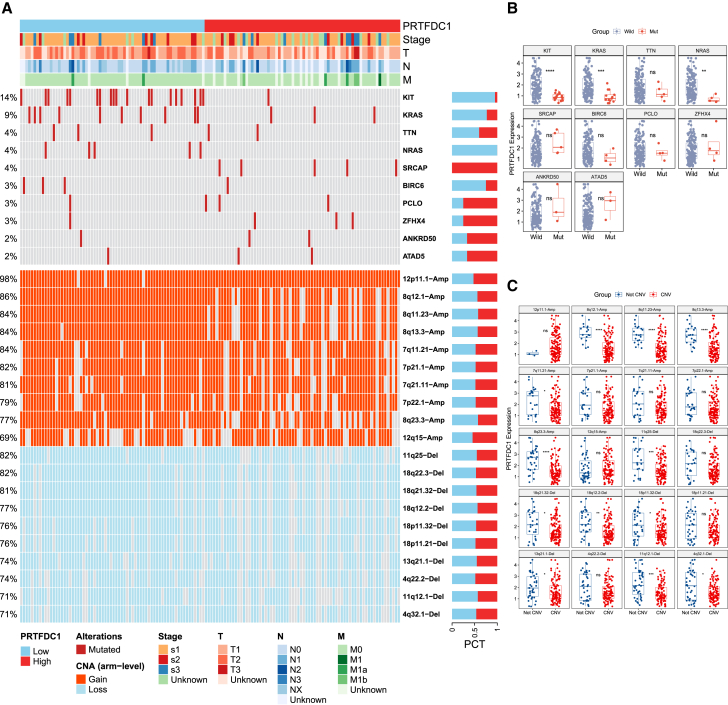
Identification of mutations and copy number variations related to PRTFDC1 (A) Mutation landscape of the top 10 frequently mutated genes (FMGs) and copy number variation (CNV) landscape of the top 10 amplified and deleted CNV chromosome segments. (B) Expression levels of PRTFDC1 in wild-type and mutant groups among the top 10 FMGs. (C) Expression levels of PRTFDC1 in variant and non-variant groups among the top 10 amplified and deleted CNV chromosome segments. ns, *p* > 0.05, ∗*p* < 0.05, ∗∗*p* < 0.01, ∗∗∗*p* < 0.001.

In the TCGA-TGCT cohort, we identified 10 frequently mutated genes (FMGs), including KIT (14%), KRAS (9%), TTN (4%), NRAS (4%), SRCAP (4%), BIRC6 (3%), PCLO (3%), ZFHX4 (3%), ANKRD50 (2%), and ATAD5 (2%; Figure 6A). We observed significant differences in the mutation frequencies of KIT, KRAS, and NRAS between the high and low PRTFDC1 expression groups (Figure S3A). Additionally, PRTFDC1 expression showed significant differences between the mutant and wild-type forms of KIT, KRAS, and NRAS (Figure 6B). We also analyzed the copy number variation (CNV) status of the top 10 frequently amplified and deleted chromosomal segments in the high and low PRTFDC1 expression groups. The results indicated that PRTFDC1 expression levels were significantly lower in the subgroups with amplifications at 8q12.1, 8q11.23, 8q13.3, 7q11.21, and 8q23.3 and deletions at 11q25, 18q21.32, 18q12.2, 18p11.32, 13q21.1, 11q12.1, and 4q32.1 compared with non-variant subgroups (Figure 6C). The amplifications at 8q12.1, 8q11.23, 8q13.3, and 8q23.3, and deletions at 11q25, 18q22.3, 18q21.32, 18q12.2, 13q21.1, and 11q12.1 showed significant differences between the high and low PRTFDC1 expression groups (Figure S3B).

### Immune infiltration and response to immunotherapy

**Figure 7:**
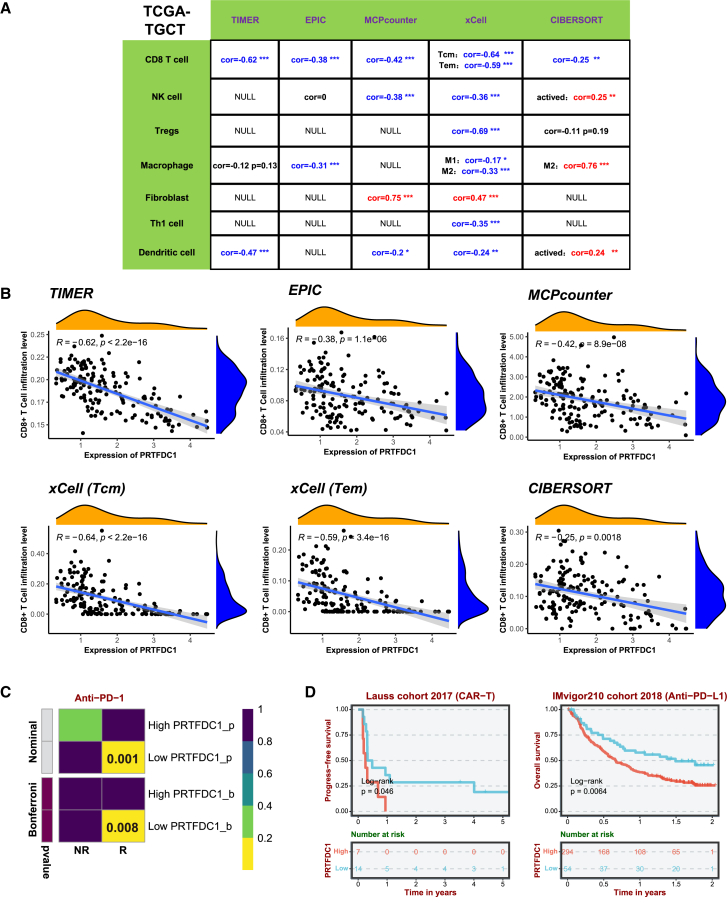
Tumor immune microenvironment and immunotherapy response of PRTFDC1 (A) Summary of the correlation analysis between PRTFDC1 expression and immune cell infiltration levels. (B) Scatterplot showing the correlation between PRTFDC1 expression and CD8^+^ T cell infiltration levels. (C) Contingency table illustrating the relationship between immunotherapy responses and PRTFDC1 groups based on the Subclass Mapping (SubMap) algorithm. (D) Kaplan-Meier survival curve comparing PRTFDC1 expression subgroups in immunotherapy cohorts (GSE100797 and IMvigor210). ∗*p* < 0.05, ∗∗*p* < 0.01, ∗∗∗*p* < 0.001.

Based on the GSEA results, we found that high PRTFDC1 expression significantly suppresses immune-related pathways. To explore this further, we analyzed the correlation between PRTFDC1 expression and immune cell infiltration scores in the TCGA-TGCT cohort using the TIMER, EPIC, MCPcounter, xCell, and CIBERSORT algorithms (Figure 7A). All five algorithms consistently showed a significant negative correlation between PRTFDC1 expression levels and CD8^+^ T cell infiltration scores (Figure 7B). This suggests that PRTFDC1 may contribute to poor prognosis by inhibiting CD8^+^ T cell infiltration in the TGCT microenvironment.

Following the identification of a potential link between PRTFDC1 and the TGCT immune microenvironment, we further investigated the potential utility of PRTFDC1 as a biomarker for immunotherapy. Subclass Mapping (SubMap) analysis revealed that patients with low PRTFDC1 expression had a higher immune response rate to anti-PD-1 therapy (*p* < 0.01; Figure 7C). Subsequently, we conducted survival analysis on two cohorts receiving chimeric antigen receptor T cell immunotherapy (CAR-T) and anti-PD-L1 immunotherapy (GSE100797 and IMvigor210) from the Biomarker Exploration for Solid Tumors (BEST) database. The results showed that patients with low PRTFDC1 expression had better prognoses, suggesting that these patients benefit more from CAR-T and anti-PD-L1 immunotherapy (Figure 7D).

### Potential drug targets

**Figure 8:**
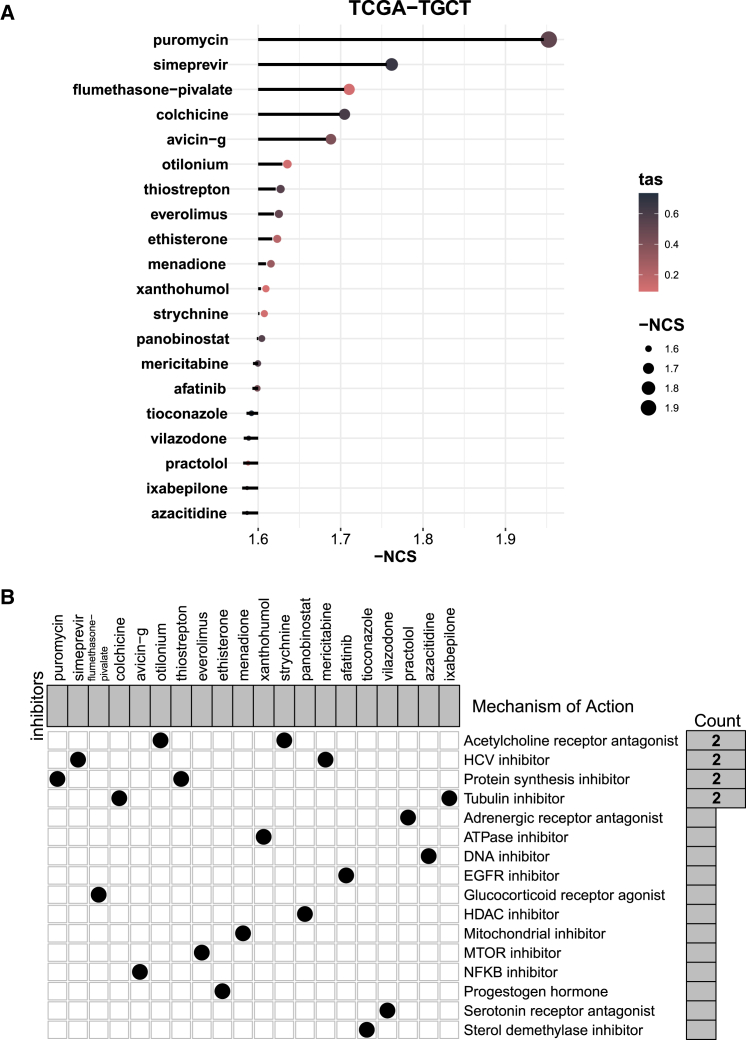
Exploration of potential drug targets related to PRTFDC1 (A) Top 20 potential therapeutic drugs highly correlated with PRTFDC1, ranked by descending NCS. (B) Heatmap illustrating the possible mechanisms of action for each compound in the CMap database.

We utilized the Connectivity Map (CMap) database to identify potential compounds targeting PRTFDC1-related pathways. We ranked perturbagens in ascending order based on their normalized connectivity scores (NCS) to identify the top 20 potential therapeutic drugs for TGCT that are highly associated with PRTFDC1. This list includes puromycin, simeprevir, flumethasone-pivalate, colchicine, avicin-g, otilonium, among others (Figure 8A). These drugs may exert their effects by inhibiting mechanisms such as acetylcholine receptors, HCV, protein synthesis, and microtubules (Figure 8B).

## Discussion

TGCT is the most common malignancy of the testis. Thanks to advancements in modern treatment methods, including the combined use of surgery, chemotherapy, and radiotherapy, most patients have a favorable prognosis. In recent years, the clinical application of immunotherapy and targeted therapy for TGCT remains in the exploratory and developmental stages, facing challenges such as uncertain treatment efficacy, resistance, side effects, and individual variability. Compared with other cancer types, TGCT generally has a lower tumor mutational burden (TMB), which results in fewer neoantigens being expressed and reduces the likelihood of immune system-mediated attacks on tumor cells.^18^ This makes TGCT less responsive to immune checkpoint inhibitor therapies.^19^ Additionally, TGCT-specific genetic mutations may impact the efficacy of both immunotherapy and targeted therapy. For example, KIT mutations, commonly found in seminomas, are relatively rare in non-seminomatous germ cell tumors, potentially affecting the effectiveness of targeted therapies.^20^ KRAS mutations may be associated with drug resistance.^21^ Therefore, there is an urgent need to identify new biomarkers to provide more accurate prognostic and immune response assessments to support clinical decision-making. In the present study, we identified a gene, PRTFDC1, associated with TGCT prognosis through bioinformatics methods and IHC, and found that it has good predictive capability for immunotherapy response in subsequent studies.

During the identification of differentially expressed genes between TGCT and normal testicular tissue, we observed an interesting phenomenon: the number of downregulated genes significantly exceeded that of upregulated genes. This could be attributed to various factors, including technical and statistical methods, as well as the biological characteristics of TGCT. Technical and statistical reasons may include the choice of data analysis and statistical methods, RNA sample degradation, and batch effects. Biological reasons may involve tumor cell apoptosis and growth inhibition, changes in transcription factor activity, genomic instability, and alterations in cellular metabolism and energy states. Consistent with previous findings by Zhang et al. on TGCT,^22^ we also observed a significantly higher number of downregulated genes compared with upregulated genes. This imbalance in differential gene expression is not only seen in TGCT but also in other types of cancers, such as breast and gastric cancer.^23^^,^^24^ In TGCTs, the dominance of downregulated genes may be associated with early growth inhibition and apoptosis of tumor cells. This phenomenon could result from the activation of tumor suppressor genes, leading to cell-cycle arrest.^17^ Additionally, tumor cells may adapt to the harsh microenvironment (such as hypoxia) by altering their metabolic pathways to reduce energy consumption and biosynthesis, which in turn leads to the downregulation of several cell metabolism-related genes.^25^ Based on these findings, potential therapeutic strategies for TGCT may include the use of activators of tumor suppressor genes or drugs that regulate cell metabolism pathways.

Subsequently, by intersecting differentially expressed genes and prognostic genes, we identified PRTFDC1 as a significant biomarker for predicting TGCT prognosis. Comprehensive analysis of independent cohorts for TGCT’s PFI and OS revealed that patients with high PRTFDC1 expression had worse overall prognosis compared with those with low expression. This observation suggests that PRTFDC1 may be a gene associated with poor prognosis. PRTFDC1 is involved in regulating the purine nucleotide salvage pathway, although its precise role in purine metabolism remains unclear.^26^ Studies have shown that, due to the increased metabolic demands of tumor cells for nucleotide synthesis and DNA replication, the purine nucleotide salvage pathway plays a critical role in maintaining nucleotide pool balance and promoting tumor growth.^27^ PRTFDC1 may exhibit different roles across various cancer types. Studies suggest that in oral squamous cell carcinoma and ovarian cancer, PRTFDC1 may have tumor-suppressive effects.^28^^,^^29^ However, high expression of PRTFDC1 is positively correlated with the triple-negative basal-like immune-suppressed breast cancer subtype (TNBC-BLIS), which is considered one of the poorest prognostic subtypes of breast cancer.^30^ In TGCT, our study found that high expression of PRTFDC1 is associated with poor prognosis, which contradicts its tumor-suppressive role in other cancers. This discrepancy may arise from the different biological functions and interaction networks of PRTFDC1 in distinct cancers. Therefore, personalized treatment strategies should be based on the specific role of PRTFDC1 in each cancer type. In TGCT or TNBC-BLIS, where PRTFDC1 may promote tumor progression, its expression could be reduced or its associated signaling pathways blocked to inhibit its function. Conversely, in cancers where PRTFDC1 acts as a tumor suppressor, strategies to enhance its expression or function should be considered to harness its potential in inhibiting tumor growth.

According to the GSEA results, high expression of PRTFDC1 activates pathways associated with cancer progression, such as the TGF-β signaling pathway, while inhibiting immune-related pathways. Additionally, we observed a negative correlation between PRTFDC1 expression and CD8^+^ T cell infiltration.CD8^+^ T cells recognize tumor-associated antigens, selectively kill tumor cells, and induce anti-tumor responses, with increased infiltration typically associated with better prognosis.^31^ Several studies have shown that in various cancers, including breast cancer, melanoma, and non-small cell lung cancer, TGF-β promotes CD8^+^ T cell exhaustion and suppresses anti-tumor immune responses, thereby facilitating tumor progression.^32^^,^^33^^,^^34^ Based on these findings, we hypothesize that PRTFDC1 may promote TGCT progression by activating the TGF-β signaling pathway, which in turn reduces CD8^+^ T cell infiltration. This finding offers new insights into the potential role of PRTFDC1 in tumor immune evasion and suggests that PRTFDC1 may be a crucial regulator of the TGCT immune microenvironment. Recently, Meng et al. proposed a stratification method for TGCT patients based on tumor immune microenvironment activation status.^35^ Their study indicated that patients in the immune-activated subgroup might respond better to anti-PD-1/PD-L1 immunotherapy. Previous research has confirmed that anti-PD-1 and PD-L1 treatments activate the immune system by inhibiting immune checkpoints, thereby killing cancer cells.^36^ SubMap analysis showed that patients with low PRTFDC1 expression responded to anti-PD-1 therapy, whereas those with high expression did not show a significant response. Additionally, in cohorts receiving CAR-T and anti-PD-L1 immunotherapy, patients with low PRTFDC1 expression had significantly better prognoses. These results suggest that patients with low PRTFDC1 expression, upregulated immune checkpoints, and increased CD8^+^ T cell infiltration are more likely to benefit from immunotherapy. In contrast, for patients with high PRTFDC1 expression, downregulated immune checkpoints, and lower CD8^+^ T cell infiltration, immunotherapy may have limited efficacy. Therefore, new treatment strategies or combination therapies, such as targeted therapies or the use of TGF-β signaling pathway inhibitors, may need to be explored.

It is well known that gene mutations are a crucial mechanism in cancer development, endowing TGCT cells with the ability to proliferate rapidly and uncontrollably, evade the immune system and other defense mechanisms, and invade other tissues.^37^ Our study found that in samples with high expression of PRTFDC1, the mutation frequencies of KIT, KRAS, and NRAS were lower. These mutated genes are common oncogenes in TGCTs and primarily promote tumor progression by activating the PI3K-AKT and RAS-MAPK signaling pathways, which stimulate cell proliferation or inhibit apoptosis.^38^^,^^39^ We hypothesize that high PRTFDC1 expression may enhance purine nucleotide reutilization, thereby supporting the tumor cells' dependence on proliferative signals. Consequently, we speculate that in the high PRTFDC1 expression group, tumor cells may be more reliant on the purine nucleotide salvage pathway to promote proliferation, rather than on the signaling pathways activated by KIT, NRAS, or KRAS mutations. In contrast, in the low PRTFDC1 expression group, tumor cells may depend more on these mutated oncogenes and their associated signaling pathways to drive proliferation and survival.

Chromosome 12p amplification is observed in almost all TGCTs and is closely associated with the development of these tumors.^37^ Additionally, studies have found that in the low PRTFDC1 expression group, the frequency of amplification in the 8q11 region is higher. Notably, the 8q11 region contains several tumor suppressor genes, such as RB1-inducible coiled-coil 1 (RB1CC1). RB1CC1 inhibits cell proliferation by regulating the stability of p53.^40^ p53, in turn, promotes peroxisomal fatty acid β-oxidation, which inhibits purine synthesis and thereby suppresses tumor growth.^41^ Based on these observations, we hypothesize that in the low PRTFDC1 expression group, 8q11 amplification may inhibit TGCT progression by activating the p53 signaling pathway, thereby suppressing purine synthesis. Our study similarly observed these chromosomal abnormalities. These results reveal a relationship between PRTFDC1 and other known oncogenic mutations and chromosomal abnormalities. These findings provide important clues for studying the specific mechanisms of PRTFDC1 in TGCTs and may offer a theoretical basis for developing new therapeutic strategies.

Finally, we investigated potential drug targets for TGCT patients and identified 20 potential TGCT therapeutic drugs related to PRTFDC1, exploring their mechanisms of action. Everolimus is a targeted drug used for the treatment of renal cell carcinoma, breast cancer, and pancreatic neuroendocrine tumors. It induces apoptosis and slows tumor cell proliferation by inhibiting the mammalian target of rapamycin (mTOR) signaling pathway.^42^^,^^43^^,^^44^ However, several phase II clinical trials have shown that everolimus monotherapy has limited efficacy in patients with cisplatin-refractory TGCTs.^45^^,^^46^ Panobinostat, a histone deacetylase inhibitor, is primarily used to treat multiple myeloma. It exerts its effects by inducing cell-cycle arrest, promoting cell differentiation and death, reducing angiogenesis, and modulating immune responses.^47^ Preclinical studies have demonstrated the effectiveness of panobinostat in treating cisplatin-resistant TGCT cell lines.^48^

This study inevitably has some limitations. Due to the relative rarity of TGCTs, the limited sample size in public databases, and potential biases from the retrospective nature of the study, future research should focus on establishing standardized data collection and detection methods. Multi-center, prospective randomized controlled trials should be designed to validate these findings and improve their generalizability. Additionally, the role of PRTFDC1 in TGCT is not fully understood and warrants further investigation through the TGCT cell lines and animal models to explore its role.

In conclusion, our study indicates that PRTFDC1 can serve as an effective and reliable novel biomarker for predicting TGCT prognosis and immunotherapy response. This finding contributes new directions and foundations for developing precision and personalized treatment strategies.

## Materials and methods

### Collection and processing of public data

We downloaded expression profiling by array and corresponding clinical data for four TGCT cohorts from the GEO database (GEO: GSE3218 (GPL96, GPL97),^49^ GSE10783 (GPL96, GPL97)^50^). The raw data were preprocessed using the Robust Multi-Array Average (RMA) method in the "affy" R package (version 1.76.0) for background correction, normalization, and log2 transformation.^51^ Based on the same platform, GSE3218-10783 (GPL96) is composed of GSE3218 (GPL96) and GSE10783 (GPL96), while GSE3218-10783 (GPL97) consists of GSE3218 (GPL97) and GSE10783 (GPL97). The ComBat function from the "sva" R package (version 3.50.0) was employed to remove batch effects (Figure S1).^52^ RNA-seq data (FPKM value, fragments per kilobase of transcript per million mapped reads, is a normalized measure of gene expression, accounting for both gene length and total read count), mutation data, and clinical data for the TGCT cohort were acquired from The Cancer Genome Atlas (TCGA-TGCT)^53^ via the UCSC Xena database.^54^ Furthermore, 48 human testicular tissue microarrays were purchased from Taibosi Biotechnology Co., Ltd. (Xi’an, China). The human tissue sample involved in the study have been approved by the Ethics Committee of Huizhou Central People's Hospital (Approval No.: No.KYLL2023106).

### Identification of differentially expressed genes

In the two merged cohorts, differentially expressed genes between tumor and normal samples were identified using the "limma" R package (version 3.58.1), with criteria set at |log2 FC| > 1.5 (FC, stands for fold change, represents the multiple change in the expression level of the target molecule under the experimental condition relative to the control condition) and adjPval <0.05.^55^

### Functional enrichment analysis

The "clusterProfiler" R package (version 4.10.0) was used to perform KEGG enrichment analysis on the differentially expressed genes to explore their potential biological functions.^56^ Pathways with *p* < 0.05 were considered significantly enriched.

### Identification of prognostic genes

Univariate Cox regression analysis was conducted using the "survival" R package (version 3.5.8) on five cohorts containing PFI or OS information (TCGA-TGCT, GSE3218 (GPL96), GSE3218 (GPL97), GSE10783 (GPL96), GSE10783 (GPL97)).^57^ Genes with a hazard ratio (HR) >1 or <1, and a *p* value <0.05, are considered associated with prognosis.

### Evaluation of differentially expressed prognostic genes

We identified the intersecting gene (PRTFDC1) by combining results from univariate Cox analysis and differential expression analysis. Using the "survminer" R package (version 0.4.9), we determined the optimal cutoff value for PRTFDC1 expression and divided the samples into high and low expression groups. Kaplan-Meier survival analysis was then performed to compare differences in PFI and OS between these groups. Additionally, multivariate Cox regression analysis was conducted to assess the prognostic value of PRTFDC1 for PFI and OS.

### Gene set enrichment analysis

To explore the biological characteristics of PRTFDC1, we calculated correlation coefficients between PRTFDC1 and other mRNAs using the Spearman correlation method. mRNAs with |Cor| >0.4 (Cor, stands for correlation, indicates the statistical relationship between PRTFDC1 expression level and the expression levels of other mRNAs) and a false discovery rate (FDR) < 0.05 were selected and ranked in descending order. The "clusterProfiler" R package was then used to perform GSEA on the ranked gene list to identify significantly enriched pathways.

### Immunohistochemistry staining

We performed IHC on testicular tissue microarrays to validate the predictive value of PRTFDC1. The antibody used was anti-PRTFDC1 (11986-1-AP, ProteinTech Group, Inc. [Wu’han, China]). The detailed experimental procedures followed our previously published protocol.^58^ The percentage of positive cells was scored as 0 (<5%), 1 (5%–25%), 2 (25%–50%), 3 (50%–75%), and 4 (>75%), and staining intensity was scored as 0 (negative), 1 (weak), 2 (moderate), and 3 (strong). The final IHC score was obtained by summing the percentage and intensity scores. The "pROC" R package (version 1.18.5) was used for ROC curve analysis to evaluate the predictive accuracy of PRTFDC1, with an ROC area under the curve (AUC) greater than 0.7 indicating good predictive performance.^59^

### Analysis of gene mutations and copy number variations

To investigate genomic mutations between the high and low PRTFDC1 expression groups, we used the "maftools" (version 2.18.0) and "ComplexHeatmap" (version 2.16.0) R packages to plot mutation waterfalls for the top 10 FMGs in the TCGA cohort.^60^^,^^61^ Additionally, we plotted CNV waterfalls for the top 10 amplified and deleted chromosomal segments in the TCGA cohort. The Wilcoxon test was used to assess differences in PRTFDC1 expression levels among different mutation and variation subgroups, and chi-square tests were conducted to analyze differences in FMGs and CNVs between PRTFDC1 expression subgroups.

### Immune infiltration and immunotherapy response

We used the "IOBR" R package (version 0.99.8) to quantify immune cell infiltration levels in the TCGA-TGCT dataset.^62^ Twenty-two immune cell infiltration scores were obtained using five algorithms, and Spearman correlation analysis was performed to assess the relationship between PRTFDC1 expression and each immune cell score. Using the SubMap algorithm,^63^ we analyzed the transcriptome expression patterns between high and low PRTFDC1 expression groups and patients responding to anti-PD-1 immunotherapy, evaluating similarity based on adjusted *p* values. In addition, we compiled two cohorts from the BEST database that received CAR-T and anti-PD-L1 immunotherapy (GSE100797, IMvigor210).^64^^,^^65^^,^^66^ Patients were grouped based on the optimal cutoff value of PRTFDC1 expression, and Kaplan-Meier survival analysis was conducted to explore the prognostic value of PRTFDC1 in immunotherapy.

### Prediction of potential therapeutic drugs

CMap is a tool used to predict the activation or inhibition effects of compounds based on gene expression profiles.^67^ We used the CLUE (https://clue.io/query/) to input 150 upregulated and 150 downregulated differentially expressed genes as the query signature and matched them with the reference profiles of perturbagens in the CMap database to calculate the NCS. By sorting the perturbagens in ascending order based on their NCS, we identified the top 20 perturbagens, which were predicted as potential therapeutic drugs for TGCTs. We further explored their possible mechanisms of action (MoA).

### Statistical analysis

Data processing and visualization were performed using R software (version 4.3.3), with some charts generated by Sangerbox (http://vip.sangerbox.com/).^68^ Correlation analysis was conducted using the Spearman method. Kaplan-Meier analysis was performed using the "survival" R package. The predictive accuracy of PRTFDC1 was evaluated based on the area under the ROC curve. The Wilcoxon rank-sum test was used to compare differences between two groups. All statistical tests were two-sided, with a *p* value <0.05 considered statistically significant.

## Data and code availability

The datasets analyzed during the current study are available in UCSC Xena (https://xenabrowser.net/datapages/;TCGA-TGCT,https://xenabrowser.net/datapages/?cohort=GDC%20TCGA%20Testicular%20Cancer%20(TGCT)&removeHub=https%3A%2F%2Fxena.treehouse.gi.ucsc.edu%3A443) and GEO(https://www.ncbi.nlm.nih.gov/geo/; GSE3218, https://www.ncbi.nlm.nih.gov/geo/query/acc.cgi; GSE10783, https://www.ncbi.nlm.nih.gov/geo/query/acc.cgi). The data generated based on the public database are available from the corresponding author upon reasonable request. The code used for analysis is available on GitHub at https://github.com/LouisK66OW/PRTFDC1.

## Acknowledgments

The authors thank the GEO and UCSC Xena for the datasets available. The research was supported by the Huizhou Science and Technology Bureau - Medical and Health Project, China (Grant No. 2022CZ010004), and the Regional Joint Fund—Regional Cultivation Project, China (Grant No. 2023A1515140040).

## Author contributions

J.C., J.L., and S.F. conceived and designed the study. P.H., Y.C., and Y.S. performed the bioinformatics analyses. H.L., X.Y., K.C., and L.Z. executed the statistical analyses. C.Z. and Z.H. conducted the experimental validation. P.H. and Y.C. drafted and revised the manuscript. All authors have read and approved the final manuscript.

## Declaration of interests

The authors declare no competing interests.

## Supplemental information

Document S1. Figures S1–S3

Document S2. Article plus supplemental information
