# Supplementary material for: Phosphoribosyl transferase domain containing 1: A prognostic biomarker in testicular germ cell tumors
Source: Mol Ther Oncol. 2025 Feb 28;33(2):200958. doi: 10.1016/j.omton.2025.200958 (PMC12001118; doi:10.1016/j.omton.2025.200958)
Supplement: Document S1. Figures S1–S3 [file mmc1.pdf]

OMTON, Volume *33*

Supplemental information

Phosphoribosyl transferase domain containing 1: A prognostic biomarker in testicular germ cell tumors

Peisheng Huang, Yihao Chen, Yongcheng Shi, Chuanfan Zhong, Huawei Lin, Xiaoxue Yu, Kai Chen, Zhuoya Huang, Le Zhang, Shumin Fang, Jianming Lu, and Jiahong Chen

Supplemental Figure

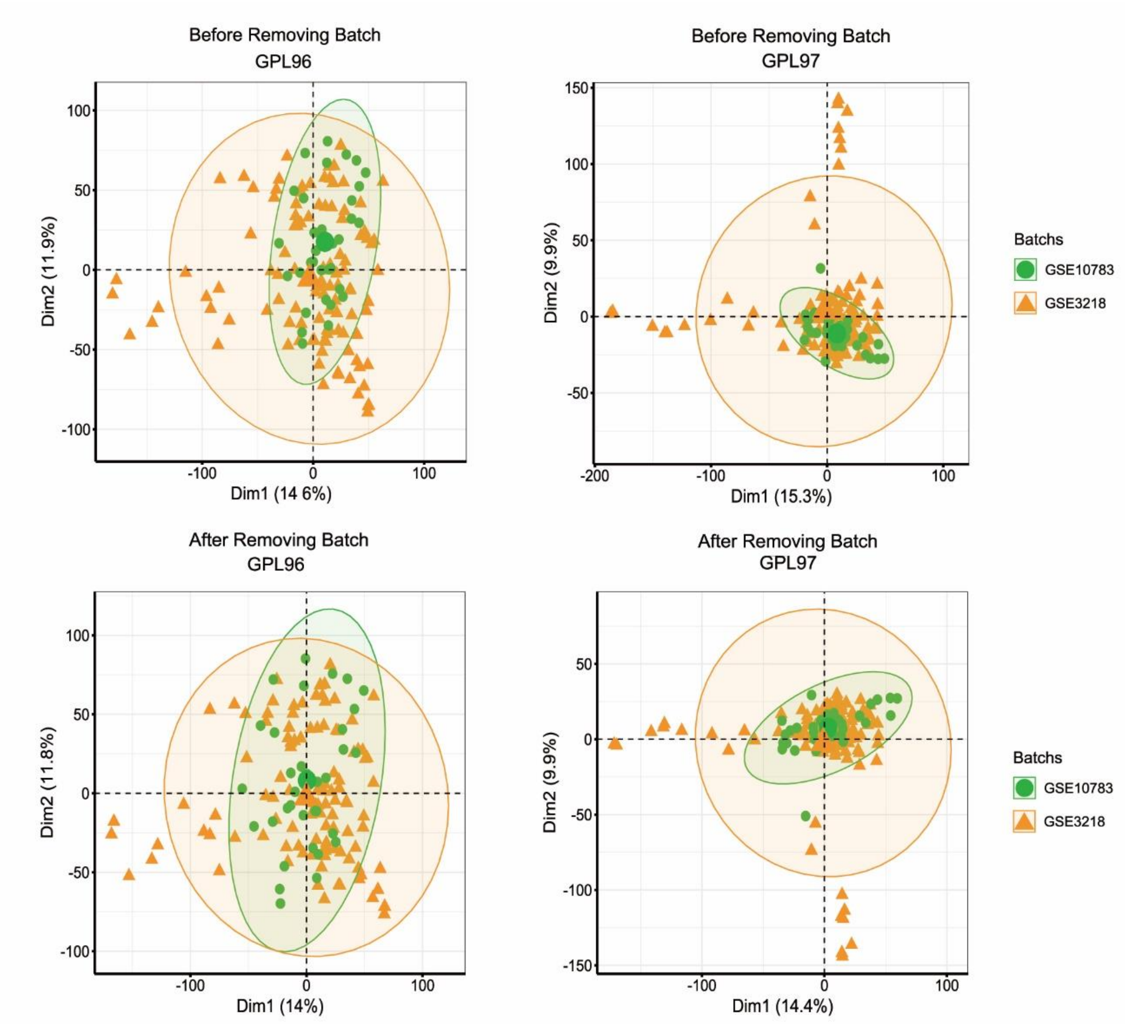

Figure S1. Batch correction results for the GSE3218 and GSE10783 (GPL96, GPL97) datasets, related to Figures 2.

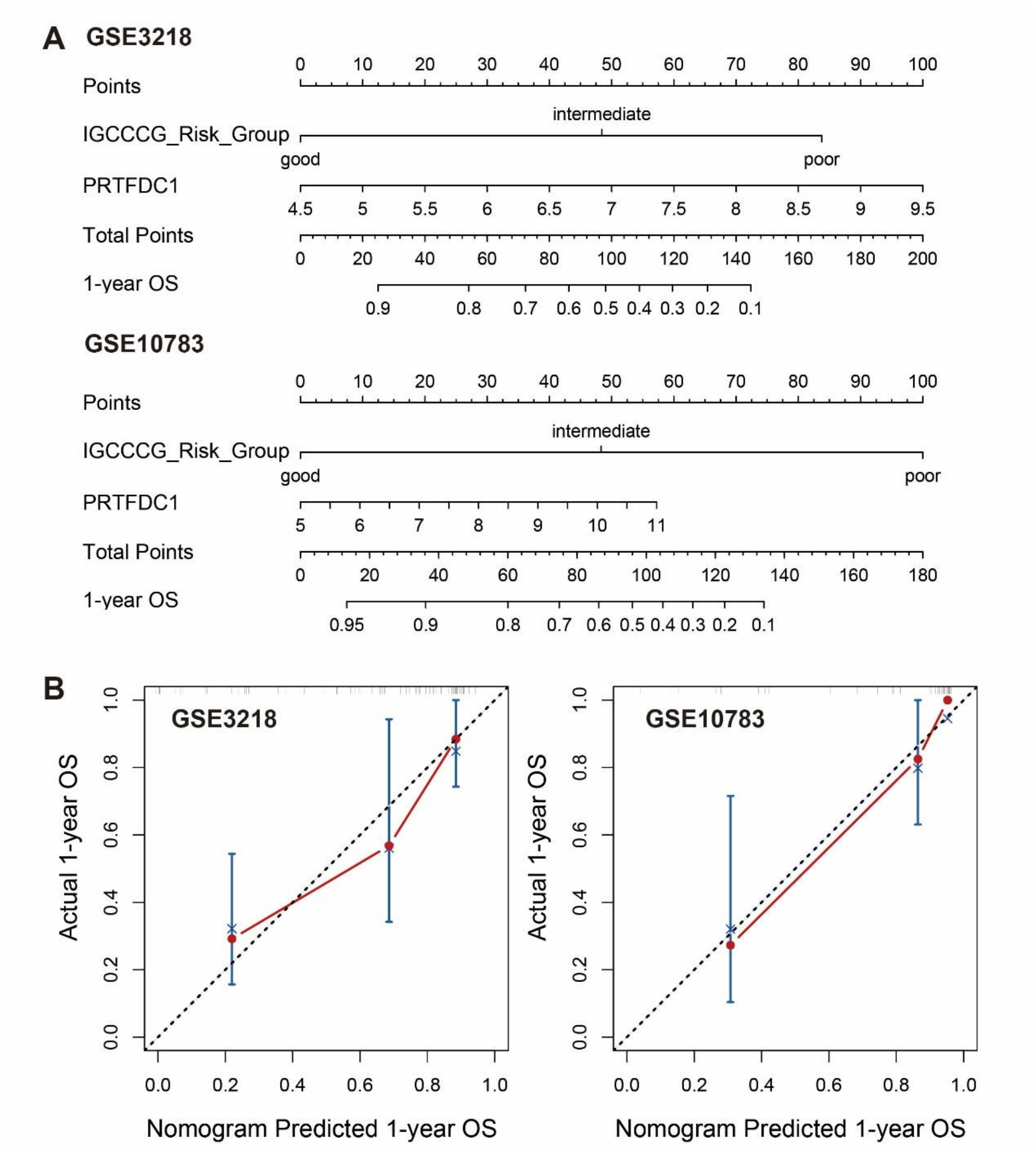

Figure S2. Nomogram Based on PRTFDC1 Expression Levels and IGCCCG Risk Group, related to Figures 3. (A) Nomogram predicting 1-year overall survival (OS) in TGCT patients from the GSE3218 and GSE10783 cohorts. (B) Calibration curve assessing the accuracy of the nomogram in predicting 1-year OS in TGCT patients from the GSE3218 and GSE10783 cohorts.

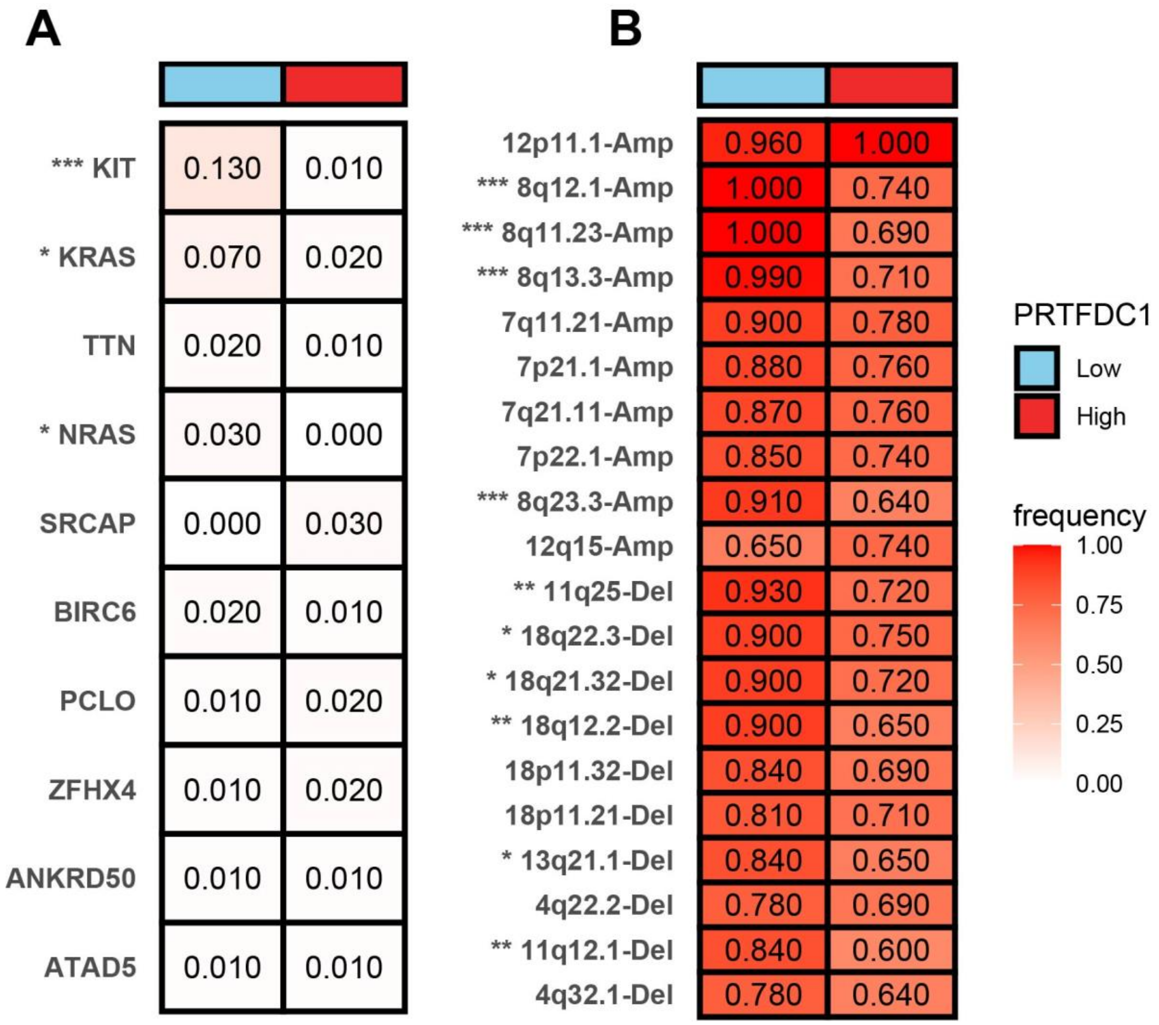

Figure S3. Analysis of PRTFDC1-related mutation and copy number variation, related to Figures 6. (A) Mutation frequencies of the top 10 FMGs between the low and high PRTFDC1 expression groups. (B) Variation frequencies of the top 10 amplified and deleted chromosome segments between the low and high PRTFDC1 expression groups. * P< 0.05, ** P < 0.01, *** P < 0.001
